# Supplementary figures and images for: Population based hospitalization burden of laboratory-confirmed hand, foot and mouth disease caused by multiple enterovirus serotypes in Southern China
Source: PLoS One. 2018 Dec 13;13(12):e0203792. doi: 10.1371/journal.pone.0203792 (PMC6292616; doi:10.1371/journal.pone.0203792)

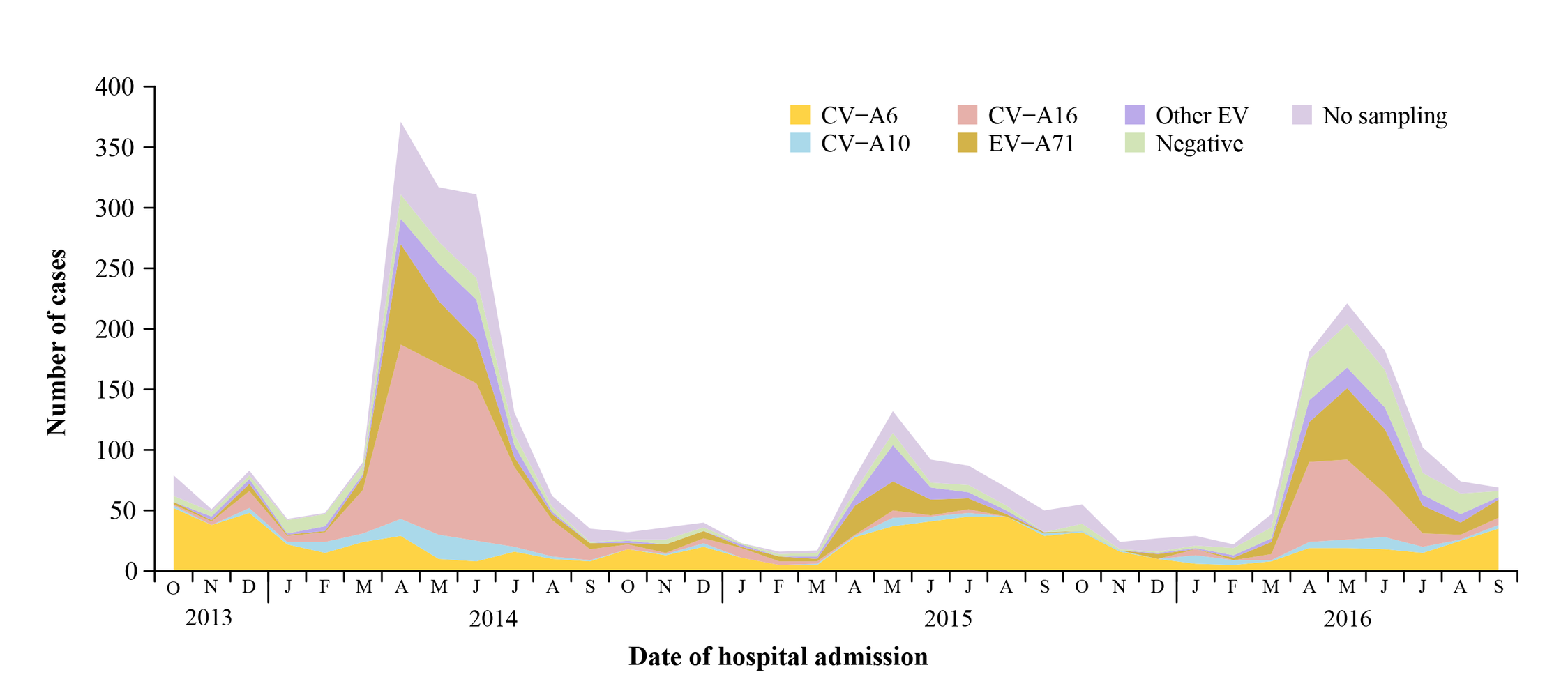

Supplement: S1 Fig — (TIF) [file pone.0203792.s001.tif]

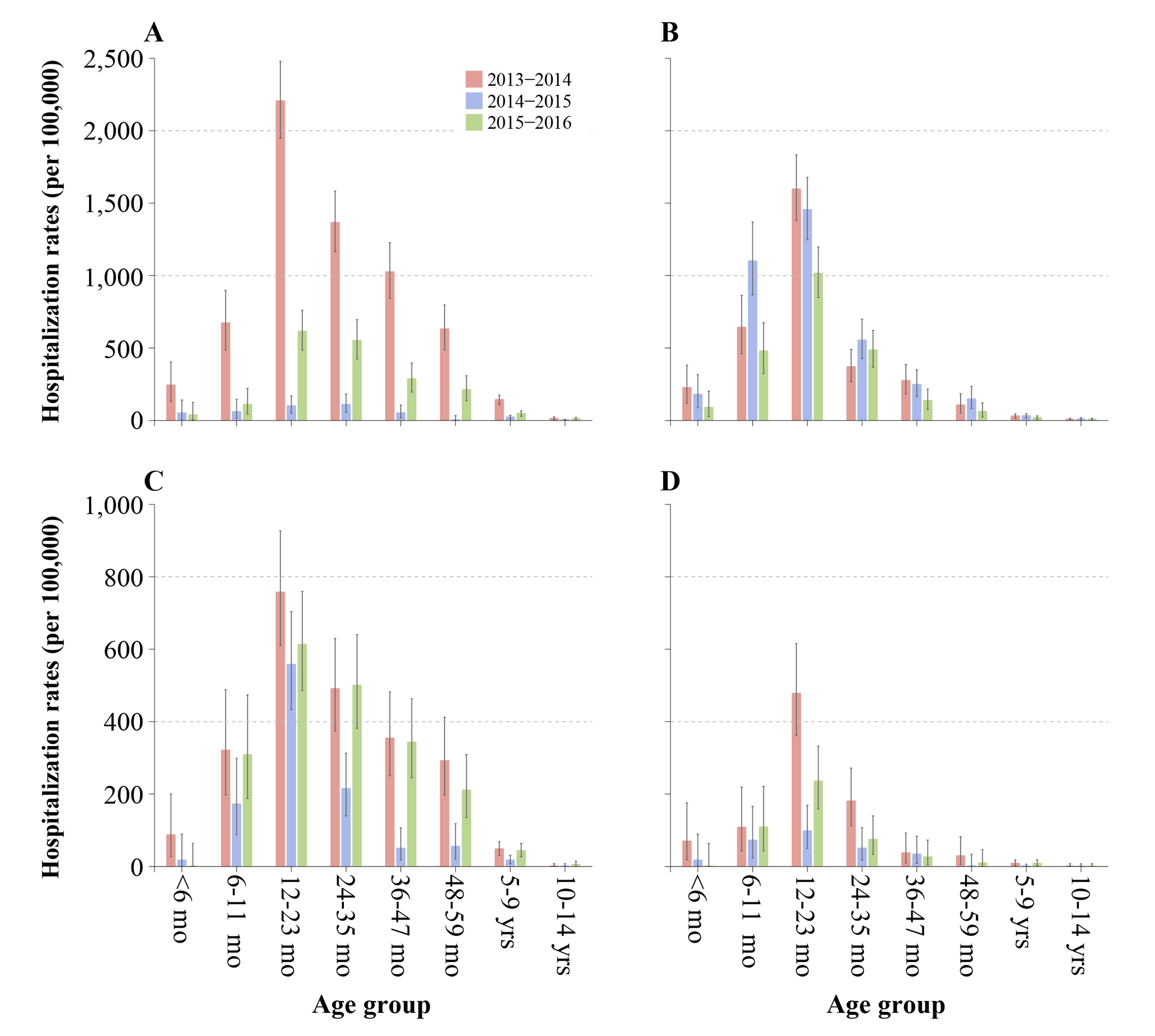

Supplement: S2 Fig — (A) Annual age-specific hospitalization rates of CV-A16-associated HFMD. (B) Annual age-specific hospitalization rates of CV-A6-associated HFMD. (C) Annual age-specific hospitalization rates of EV-A71-associated HFMD. (D) Annual age-specific hospitalization rates of CV-A10-associated HFMD. (TIF) [file pone.0203792.s002.tif]
