# Supplementary material for: Population based hospitalization burden of laboratory-confirmed hand, foot and mouth disease caused by multiple enterovirus serotypes in Southern China
Source: PLoS One. 2018 Dec 13;13(12):e0203792. doi: 10.1371/journal.pone.0203792 (PMC6292616; doi:10.1371/journal.pone.0203792)
Supplement: S1 Table — (DOCX) [file pone.0203792.s003.docx]

**S1 Table. Primers and probes used in the real time RT-PCR.**

| Primer | Sequence (5’-3’) |
| --- | --- |
| EV71-F | TGATTGAGACACGSTGTGTYCTTA |
| EV71-R | CCCGCTCTGCTGAAGAAACT |
| EV71-P | FAM-TCGCACAGCACAGCTGAGACCACTC-BHQ1 |
| CA16-F | GGGAATTTCTTTAGCCGTGC |
| CA16-R | CCCATCAARTCAATGTCCC |
| CA16-P | FAM-ACAATGCCCACCACGGGTACACA-BHQ1 |
| CA6-F | CAAGCTGCAGAAACGGGAG |
| CA6-R | GCTCCACACTCGCCTCATT |
| CA6-P | FAM-ACCCCGTTTCGATTCATCACACA-BHQ1 |
| pan-enterovirus -F | CCCTGAATGCGGCTAAT |
| pan-enterovirus -R | ATTGTCACCATAAGCAGCC |
| pan-enterovirus -P | FAM-CGGAACCGACTACTTTGGGT-3'-TAMRA |

Note： F: Forward primer, R: Reverse primer, P: probe primer.

Briefly, the reactions of the real time RT-PCR were run under the following conditions: incubation at 50 °C for 30 min, initial denaturation at 94 °C for 2 min, followed by 45 cycles of denaturation at 94 °C for 10 s, annealing at 60 °C for 20 s, and extension at 68 °C for 20 s.
