# Supplementary material for: Population based hospitalization burden of laboratory-confirmed hand, foot and mouth disease caused by multiple enterovirus serotypes in Southern China
Source: PLoS One. 2018 Dec 13;13(12):e0203792. doi: 10.1371/journal.pone.0203792 (PMC6292616; doi:10.1371/journal.pone.0203792)
Supplement: S2 Table — (DOCX) [file pone.0203792.s004.docx]

**S2 Table. Primers used in nested RT-PCR**

Primers used in the nested RT-PCR to amplify VP1 regions

| Primer | Sequence (5’-3’) | Note | Target region |
| --- | --- | --- | --- |
| SO224 | GCIATGYTIGGIACICAYRT | 1^st^ round RT-PCR | VP1 |
| SO222 | CICCIGGIGGIAYRWACAT |  |  |
| AN32 | GTYTGCCA |  |  |
| AN33 | GAYTGCCA |  |  |
| AN34 | CCRTCRTA |  |  |
| AN35 | RCTYTGCCA |  |  |
| AN89 | CCAGCACTGACAGCAGYNGARAYNGG | 2^nd^ round PCR |  |
| AN88 | TACTGGACCACCTGGNGGNAYRWACAT |  |  |
| AN232 | CCAGCACTGACAGCA | Sequencing |  |
| AN233 | TACTGGACCACCTGG |  |  |

The laboratory procedures of nested PCRs to amplify a portion of the VP1 region: The first-round PCR was performed with primers including mix AN32, AN33, AN34, AN35, 224 and 222 using SuperScript III one-step RT-PCR with Platinum Taq DNA Polymerase kit (Invitrogen, Carlsbad, CA), while AN88 and AN89 primers were used for the second round of semi-nested PCR (Supplementary Table 2). The products were gel purified and subjected to DNA sequencing. Identification of enterovirus serotypes was performed using BLAST analysis (http://www.ncbi.nlm.gov/BLAST).

Primers used in the nested RT-PCR to amplify VP4-VP2 regions

| Primer | Sequence (5’3’) | Note | | Target region |
| --- | --- | --- | --- | --- |
| 458-F | CCGGCCCCTGAATGYGGCTAA | forward | 1^st^ round RT-PCR | VP4-VP2 |
| HEVA_VP4_1217a | AYTGNGCRTTYTGNCCRAANACNCC | reverse |  |  |
| HEVB_VP4_1215a | CATRTTYTGNSCRAANARNCCYA |  |  |  |
| HEVC_VP4_1214a | ACATRTTYTGNCCRAANADNCCCAT |  |  |  |
| 547-F | ACCRACTACTTTGGGTGTCCGTG | forward | 2^nd^ round RT-PCR |  |
| HEVA_VP4_1178a | TCNGGRAAYTTCCARTACCANCC | reverse |  |  |
| HEVB_VP4_1178a | TCNGGNARYTTCCACCACCANCC |  |  |  |
| HEVC_VP4_1178a | TCNGGYARYTTCCACCACCANCC |  |  |  |

The selecting rule of RT-PCR will abide by the strategy: we will use HEVA_VP4 primers for conducting the experiments firstly, if negative, we will use HEVB_VP4 primers; if negative again, we will use HEVC_VP4 primers.

All RT-PCR will conduct in the same condition. First round amplification was performed in a 50 μl volume with the first-round primers using SuperScript III One-Step RT-PCR kit. Cycling parameters for the first-round of RT-PCR, starting with a 42 °C incubation for 60 min and then initial denaturation at 95 °C for 3 min, 25 cycles of denaturation at 94 °C for 30 s, annealing at 50 °C for 30 s, and extension at 72 °C for 30 s, final extension at 72 °C for 10 min. Exactly 1 μl of the first-round product was used as the template DNA for the second-round RT-PCR and was amplified using the second round primers. The second-round PCR conditions were the same as described above (25 μl). Then sequencing results were used in a BLAST search against the GenBank database.
