# Supplementary material for: Population based hospitalization burden of laboratory-confirmed hand, foot and mouth disease caused by multiple enterovirus serotypes in Southern China
Source: PLoS One. 2018 Dec 13;13(12):e0203792. doi: 10.1371/journal.pone.0203792 (PMC6292616; doi:10.1371/journal.pone.0203792)
Supplement: S3 Table — (DOCX) [file pone.0203792.s005.docx]

**S1 Table. The age profile of HFMD-associated hospitalizations stratified by enterovirus serotype in 6 surveillance hospitals in Anhua County, China, October 2013 - September 2016.**

| Serotype | <6 mo | 6-11 mo | 12-23 mo | 24-35 mo | 36-47 mo | 48-59 mo | 5-9 yrs | 10-14 yrs |
| --- | --- | --- | --- | --- | --- | --- | --- | --- |
| EV-A71 | 5 (9%) | 44 (17%) | 187 (18%) | 115 (22%) | 68 (25%) | 51 (31%) | 43 (25%) | 1 (7%) |
| CV-A16 | 15 (28%) | 45 (17%) | 289 (27%) | 195 (37%) | 116 (43%) | 72 (44%) | 80 (47%) | 7 (47%) |
| CV-A6 | 22 (42%) | 123 (47%) | 391 (37%) | 133 (25%) | 57 (21%) | 28 (17%) | 25 (15%) | 6 (40%) |
| CV-A10 | 4 (8%) | 16 (6%) | 80 (8%) | 30 (6%) | 9 (3%) | 3 (2%) | 7 (4%) | 0 (0%) |
| Other EV | 7 (13%) | 32 (12%) | 113 (11%) | 52 (10%) | 22 (8%) | 8 (5%) | 15 (9%) | 1 (7%) |
| Total | 53 (100%) | 260 (100%) | 1,060 (100%) | 525 (100%) | 272 (100%) | 162 (100%) | 170 (100%) | 15 (100%) |

^*^Other EV includes CV-A2 (31), CV-A4 (75), CV-A5 (8), CV-A8 (40), CV-B2 (6), CV-B3 (2), CV-B4 (2), CV-B5 (8), CV-A9 (2), E-3 (1), E-9 (5), E-16 (1), E-18 (12), E-25 (1), E-30 (1), coinfection (23 EV-A71 & CV-A6, 19 EV-A71 & CV-A16, and 9 CV-A16 & CV-A6), and un-serotype (4).
