# Supplementary material for: Population based hospitalization burden of laboratory-confirmed hand, foot and mouth disease caused by multiple enterovirus serotypes in Southern China
Source: PLoS One. 2018 Dec 13;13(12):e0203792. doi: 10.1371/journal.pone.0203792 (PMC6292616; doi:10.1371/journal.pone.0203792)
Supplement: S4 Table — (DOCX) [file pone.0203792.s006.docx]

**S2 Table.** **Estimated hospitalization rates associated with HFMD by age group in Anhua County, China, October 2013 - September 2016.**

| Age group | Year ^a^ | Population size of residents | No. of HFMD-associated hospitalizations in 3 township level hospitals | No. of Total hospitalizations in 3 township level hospitals ^b^ | Proportion of hospitalizations associated with HFMD | No. of total hospitalizations in 23 township level hospitals ^c^ | No. of HFMD-associated hospitalizations in 23 township level hospitals | No. of HFMD-associated hospitalizations in three county-level hospitals | No. of HFMD-associated hospitalizations in Anhua County | Hospitalization rates of HFMD per 100,000 |
| --- | --- | --- | --- | --- | --- | --- | --- | --- | --- | --- |
|  |  | **A** | **B** | **C** | **D=B/C** | **E** | **F=E*D** | **G** | **H=F+G** | **I=H/A *100,000** |
| <6 mo | 2013-2014 | 5,828 | 0 | 8 | 0.000 | 47 | 0 | 41 | 41 | 704 |
|  | 2014-2015 | 6,223 | 0 | 6 | 0.000 | 34 | 0 | 21 | 21 | 337 |
|  | 2015-2016 | 5,777 | 0 | 10 | 0.000 | 60 | 0 | 12 | 12 | 208 |
| 6-11 mo | 2013-2014 | 6,572 | 1 | 37 | 0.027 | 191 | 5 | 145 | 150 | 2,282 |
|  | 2014-2015 | 7,018 | 0 | 29 | 0.000 | 165 | 0 | 118 | 118 | 1,681 |
|  | 2015-2016 | 6,514 | 0 | 44 | 0.000 | 217 | 0 | 88 | 88 | 1,351 |
| 12-23 mo | 2013-2014 | 12,170 | 9 | 102 | 0.088 | 609 | 54 | 667 | 721 | 5,924 |
|  | 2014-2015 | 12,400 | 7 | 88 | 0.080 | 498 | 40 | 296 | 336 | 2,710 |
|  | 2015-2016 | 13,241 | 8 | 115 | 0.070 | 720 | 50 | 398 | 448 | 3,383 |
| 24-35 mo | 2013-2014 | 12,256 | 3 | 80 | 0.038 | 591 | 22 | 332 | 354 | 2,888 |
|  | 2014-2015 | 12,170 | 1 | 68 | 0.015 | 534 | 8 | 128 | 136 | 1,118 |
|  | 2015-2016 | 12,228 | 5 | 92 | 0.054 | 647 | 35 | 229 | 264 | 2,159 |
| 36-47 mo | 2013-2014 | 11,056 | 4 | 70 | 0.057 | 558 | 32 | 183 | 215 | 1,945 |
|  | 2014-2015 | 12,256 | 2 | 71 | 0.028 | 540 | 15 | 45 | 60 | 490 |
|  | 2015-2016 | 11,999 | 1 | 69 | 0.014 | 575 | 8 | 116 | 124 | 1,033 |
| 47-59 mo | 2013-2014 | 10,668 | 3 | 63 | 0.048 | 487 | 23 | 102 | 125 | 1,172 |
|  | 2014-2015 | 11,056 | 0 | 55 | 0.000 | 381 | 0 | 26 | 26 | 235 |
|  | 2015-2016 | 11,938 | 0 | 71 | 0.000 | 593 | 0 | 78 | 78 | 653 |
| 5-9 yrs | 2013-2014 | 55,097 | 5 | 185 | 0.027 | 1,343 | 36 | 117 | 153 | 278 |
|  | 2014-2015 | 55,581 | 3 | 134 | 0.022 | 1,072 | 24 | 21 | 45 | 81 |
|  | 2015-2016 | 54,415 | 0 | 236 | 0.000 | 1,614 | 0 | 91 | 91 | 167 |
| 10-14 yrs | 2013-2014 | 47,468 | 0 | 95 | 0.000 | 632 | 0 | 9 | 9 | 19 |
|  | 2014-2015 | 48,346 | 0 | 86 | 0.000 | 585 | 0 | 4 | 4 | 8 |
|  | 2015-2016 | 48,736 | 1 | 104 | 0.010 | 679 | 7 | 6 | 13 | 27 |
| Total | 2013-2014 | 161,115 | 25 | 640 | 0.039 | 4,458 | 174 | 1,596 | 1,770 | 1,090 |
|  | 2014-2015 | 164,015 | 13 | 537 | 0.024 | 3,809 | 92 | 659 | 751 | 458 |
|  | 2015-2016 | 164,848 | 15 | 741 | 0.020 | 5,105 | 103 | 1,018 | 1,121 | 680 |

^a.^ 2013-2014: from October 1, 2013 to September 30, 2014; 2014-2015: from October 1, 2014 to September 30, 2015; 2015-2016: from October 1, 2015 to September 30, 2016.

^b.^ No. of total hospitalizations in 3 township level hospitals from October 2013 to September 2014 = (No. of total hospitalizations in 3 township level hospitals form October 2014 to September 2015 + No. of total hospitalizations in 3 township level hospitals form October 2015 to September 2016) / 2.

^c.^ No. of total hospitalizations in 23 township level hospitals from October 2013 to September 2014 = (No. of total hospitalizations in 23 township level hospitals form October 2014 to September 2015 + No. of total hospitalizations in 23 township level hospitals form October 2015 to September 2016) / 2.
